# La tuberculose de la paroi thoracique : À propos d'un cas au CHU Joseph Ravoahangy Andrianavalona, Antananarivo, Madagascar

**DOI:** 10.48327/mtsibulletin.2021.154

**Published:** 2021-09-23

**Authors:** R.N.A.L Rakotorahalahy, S. Randrianandrasana, T. Rajaobelison, R.A. Ralaivao, L. Ramiliarijaona, A.J.C Rakotoarisoa

**Affiliations:** 1 Service de chirurgie cardio-vasculaire, Centre hospitalier universitaire Joseph Ravoahangy Andrianavalona, Ampefiloha, BP 4150,101 Antananarivo Madagascar; 2 Service d'anatomie pathologique du Centre hospitalier universitaire Joseph Ravoahangy Andrianavalona, Ampefiloha, BP 4150,101 Antananarivo Madagascar; 3 Faculté de médecine d'Antananarivo, BP 375, Antananarivo Madagascar

**Keywords:** Tuberculose, Paroi thoracique, Diagnostic, Traitement, Hôpital, Antananarivo, Madagascar, Océan Indien, Tuberculosis, Chest wall, Diagnosis, Therapy, Hospital, Antananarivo, Madagascar, Indian Ocean

## Abstract

**Introduction/justification:**

La tuberculose est un problème de santé publique. Les présentations extra-pulmonaires sont rares et de diagnostic parfois délicat. Nous rapportons un cas de tuberculose de la paroi thoracique chez un homme de 28 ans afin de discuter le mécanisme, les problèmes diagnostique et thérapeutique posés par cette localisation rare.

**Description:**

Le patient présentait une tuméfaction pariétale découverte à l'issue d'un effort physique. L'imagerie était en faveur d'un abcès ou d'un hématome infecté. La chirurgie d'exérèse a permis d’établir l'origine tuberculeuse qui a été confirmée par l'histologie. Un traitement antituberculeux institué pendant 9 mois a complété le traitement chirurgical et conduit à la guérison du patient.

**Discussion/conclusion:**

La rareté de cette localisation de la tuberculose doit être recherchée. Un traitement étiologique doit être institué et un suivi régulier programmé pour éviter les récidives.

## Abstract (en)

**Background/rationale:**

Tuberculosis is a public health issue. Extra-pulmonary presentations are rare and sometimes hard to diagnose and manage. Here, we report a case of tuberculosis of the chest wall in a 28-year-old man in order to discuss the mechanism, diagnostic and therapeutic problems posed by this rare localization.

**Description:**

The patient presented with wall swelling discovered after physical effort. Imaging was in favor of an abscess or infected hematoma. The excisional surgery established the tuberculous origin which was confirmed by histology. Anti-tuberculosis treatment during 9 months completed the surgical treatment and led to the patient's recovery.

**Discussion/conclusion:**

The rarity of this localization of tuberculosis should be investigated. Etiological treatment must be instituted and regular monitoring scheduled to avoid recurrence.

**Conclusion:**

Direct-acting antivirals are effective and characterized by good tolerance in these Malagasy hepatitis C patients.

## Introduction

À l'heure actuelle, la tuberculose constitue encore un problème de santé publique en particulier dans les pays en voie de développement [9, 10]. La tuberculose extra-pulmonaire, localisée dans la paroi thoracique, est rare et représente moins de 5% des tuberculoses ostéo-articulaires et de 0,1 % de toutes les localisations [7, 9, 10].

Notre but est de rapporter un cas que nous avons observé et de discuter du diagnostic et de la prise en charge dans le contexte malgache.

## Cas clinique

**Figure 1:**
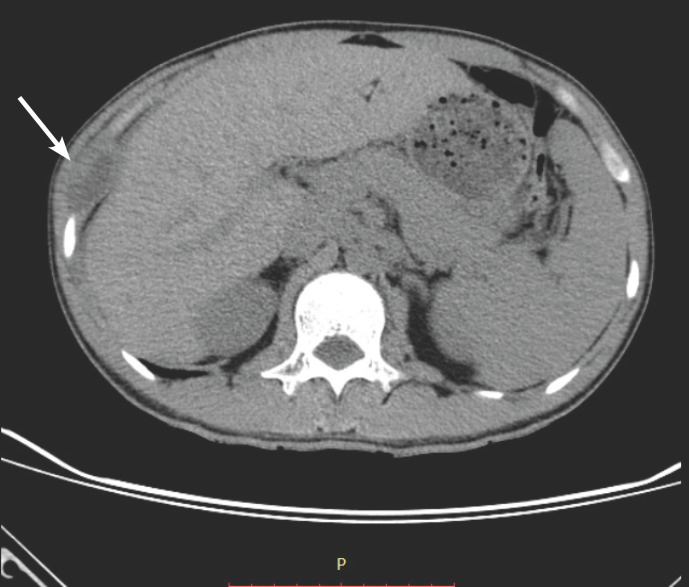
Scanner thoracique montrant la lésion en regard de la neuvième côte Chest CT scan showing the lesion over the ninth rib

Mr R.-J., âgé de 28 ans, sans antécédents particuliers ni contage tuberculeux connu, présente depuis un mois avant sa consultation, dans le service de chirurgie cardiovasculaire du CHU Joseph Ravoahangy Andrianavalona, Antananarivo, Madagascar, une tuméfaction douloureuse au niveau de la paroi thoracique latérale droite, en regard du 9^e^ espace intercostal, sans fièvre, augmentant progressivement de volume. Au moment de déplacer un objet lourd, environ une semaine avant l'apparition de la tuméfaction, le patient a senti un craquement suivi d'une douleur vive dans la région où se situe la tuméfaction. La douleur a disparu après une prise d'anti-inflammatoire, mais est réapparue après quelques jours. L'examen physique retrouvait un patient en bon état général, apyrétique présentant une masse pariétale latérale thoracique droite de 5 cm de grand axe, de consistance rénitente, sans signes inflammatoires locaux. Les aires ganglionnaires étaient libres et le reste de l'examen était sans particularité. Le bilan sanguin retrouvait une leucocytose à 8 660 éléments/mm^3^ avec des lymphocytes à 13 %, une hémoglobine à 16,2 g/dl et une vitesse de sédimentation à 20 mm à la première heure. La recherche de bacilles tuberculeux dans les crachats et la sérologie de VIH étaient revenues négatives. L'intradermoréaction (IDR) à la tuberculine était de 7 mm de diamètre. L’échographie de la tuméfaction était en faveur d'un abcès pariétal. La radiographie du thorax et la tomodensitométrie thoracique confirmaient la présence d'une formation liquidienne hétérogène mesurant 39 mm de diamètre en antéropostérieur pour 22 mm de large et 52 mm de hauteur, entourée d'une coque épaisse et contenant des logettes internes, compatible avec un abcès au niveau de la paroi thoracique latérale droite, dans le 9^e^ espace intercostal avec réaction du périoste de l'arc antérieur de la 9^e^ côte correspondante (Fig. 1). On notait également une opacité micronodulaire de répartition en arbre en bourgeon du lobe moyen et du segment supérieur du lobe inférieur du poumon droit, en rapport avec des foyers de pneumopathie probablement bacillaire.

Considéré comme un hématome infecté post traumatique, une mise à plat, avec issue de pus et de nécrose caséeuse, ainsi qu'un effondrement des logettes associé à une exérèse de la coque et du périoste, a été pratiquée afin d’éviter une brèche pleurale. Un drain lamellaire a ensuite été posé.

**Figure 2:**
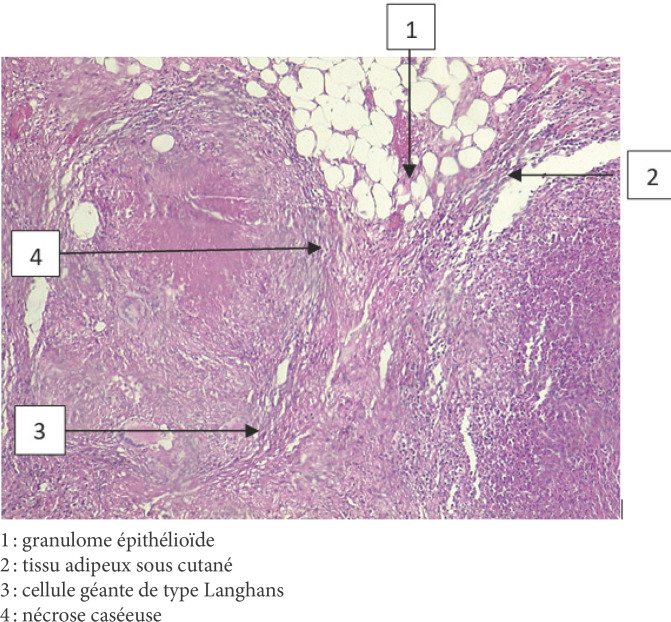
Vue microscopique (x100, coloration hématéine éosine) de la pièce biopsique de la paroi thoracique à l'examen anatomopathologique 1: granulome épithélioïde 2: tissu adipeux sous cutané 3: cellule géante de type Langhans 4: nécrose caséeuse Microscopic view (xl00, hematein-eosin staining) of the biopsy specimen of the chest wall on pathological examination

L'examen bactériologique direct et après culture, du liquide, réalisé à deux reprises, était stérile. L'examen anatomopathologique de la coque et le GeneXpert du pus étaient en faveur d'une tuberculose (Fig. 2). Le patient a été traité comme tel, selon le protocole du programme national contre la tuberculose (PNLT) à Madagascar, avec une quadrithérapie (ethambutol, rifampicine, isoniazide et pyrazinamide) suivie d'une bithérapie (isoniazide et rifampicine), pour une durée totale de 9 mois.

La plaie opératoire est restée béante pendant six semaines avec persistance de suppurations de faible quantité et n'a cicatrisé qu'après huit semaines de polychimiothérapie antituberculeuse. L'examen clinique et radiologique de contrôle était satisfaisant avec disparition de la douleur, la plèvre et le parenchyme pulmonaire sans anomalie visible. Le traitement antituberculeux a été bien toléré. Le patient, par la suite, a été suivi régulièrement et après 30 mois de surveillance, aucune récidive, clinique ou radiologique, n'a été détectée.

## Discussion

La plupart des foyers de tuberculose extrapulmonaire sont localisés au niveau de la plèvre et des ganglions [7, 9, 10]. Forme inhabituelle et rare, la tuberculose de la paroi thoracique, peut être due à une inoculation directe transcutanée, à un drainage lymphatique à partir d'une plèvre infectée ou par contigüité, à une adénite de la paroi thoracique ou encore par voie hématogène à partir d'un foyer pulmonaire [3, 12]. Ces deux derniers mécanismes peuvent être à l'origine de cette lésion pour notre patient. Comme tous les abcès, une rupture vers la cavité thoracique, une fistulisation cutanée et une dissémination loco-régionale ou à distance sont possibles [3, 4].

La manifestation clinique de cette maladie est non spécifique et se résume à une douleur, une tuméfaction pariétale, parfois une fièvre. Elle pose souvent un problème diagnostique et thérapeutique entre une tumeur et les autres abcès pariétaux à pyogènes ou infection à actinomycètes [1, 3, 4, 8]. Les signes du début sont souvent discrets d'où la découverte tardive au stade de collection. Dans notre cas, une notion de facteur déclenchant, un déplacement d'un objet lourd, signalée par le patient a égaré le diagnostic avant l'intervention chirurgicale.

La taille de la tuméfaction est variable ainsi que sa consistance. Elle est douloureuse, plus ou moins dure et parfois rénitente, mais ne présente pas dans certains cas, comme dans notre observation, de chaleur locale ou de rougeur. Les différentes étiologies d'un abcès froid de la paroi thoracique sont à éliminer devant ce tableau clinique. Les lésions sont généralement isolées, mais chez certains patients, de multiples lésions ont été trouvées dans deux ou plusieurs sites thoraciques ou extra thoraciques [6]: dans notre cas, la lésion était unique.

L'imagerie oriente le diagnostic et permet de mesurer la taille, de préciser la topographie et l'aspect de la tuméfaction, de voir s'il y a des lésions associées et le rapport avec les éléments de voisinage [1, 8, 11]. Dans notre cas, elle n'a pas permis d'envisager l’étiologie tuberculose qui a été une découverte histologique.

Le bilan sanguin n'est pas spécifique. La bactériologie est rarement positive [7, 9, 10]. L'examen anatomopathologique de la coque et le GenExpert confirment le diagnostic [7], comme chez notre patient.

Dans les zones à forte endémie tuberculeuse, la majorité des IDR positives sont le témoin d'une authentique infection tuberculeuse, par contact direct avec M. tuberculosis. Par conséquent, le critère de positivité en faveur d'une infection tuberculeuse peut être estimé à un diamètre ≥ à 5 mm [5]. L'IDR à la tuberculine de notre patient était de 7 mm.

Sur le plan thérapeutique, l'exérèse chirurgicale et totale de la lésion est le geste idéal lorsque cela est possible [2]. Certains auteurs décrivent même une exérèse large de la paroi thoracique emportant les côtes touchées par la lésion tuberculeuse puis la mise en place d'une prothèse pariétale [1, 2, 11]. Pour d'autres, une mise à plat ou une simple ponction du contenu, comme pour un abcès, peut suffire [1, 2]. Notre patient a bénéficié d'une exérèse de l'enveloppe de l'abcès et du périoste ainsi qu'un drainage pariétal, après évacuation de l'abcès. Le traitement chirurgical local doit toujours être associé aux antituberculeux, d'une durée de 9 mois dans le cas de notre patient.

Une surveillance régulière doit être proposée pour les patients, même après arrêt du traitement, car d'après la littérature une récidive est possible [1, 2, 11].

## Conclusion

La localisation de la tuberculose, au niveau de la paroi thoracique est très rare et de diagnostic difficile, mais il faut la rechercher, surtout dans les pays endémiques de tuberculose comme Madagascar pour éviter l’évolution de la maladie vers une complication. Le traitement n'est pas encore codifié, mais nécessite d'associer le traitement antituberculeux et la chirurgie. Dans certains cas, comme notre patient, la chirurgie permet, à la fois, le diagnostic et l'orientation du traitement.

## Conflits D'intérêts

L'auteur déclare ne pas avoir de conflit d'intérêt.
